# Characterization of ARF-BP1/HUWE1 Interactions with CTCF, MYC, ARF and p53 in MYC-Driven B Cell Neoplasms

**DOI:** 10.3390/ijms13056204

**Published:** 2012-05-21

**Authors:** Chen-Feng Qi, Yong-Soo Kim, Shao Xiang, Ziedulla Abdullaev, Ted A. Torrey, Siegfried Janz, Alexander L. Kovalchuk, Jiafang Sun, Delin Chen, William C. Cho, Wei Gu, Herbert C. Morse

**Affiliations:** 1 Laboratory of Immunogenetics, National Institute of Allergy and Infectious Diseases, National Institutes of Health, Rockville, MD 20852, USA; E-Mails: kimyongs@mail.nih.gov (Y.-S.K.); xiangshao@gmail.com (S.X.); zabdul@mail.nih.gov (Z.A.); kovalcha@niaid.nih.gov (A.L.K.); sunj7@mail.nih.gov (J.S.); 2 Taussig Cancer Institute, Cleveland Clinic Foundation, Cleveland, OH 44195, USA; 3 Comparative Medicine Branch, National Institute of Allergy and Infectious Diseases, National Institutes of Health, Bethesda, MD 20892, USA; E-Mail: ttorrey@niaid.nih.gov; 4 Department of Pathology, Carver College of Medicine, University of Iowa, Iowa City, IA 52242, USA; E-Mail: siegfried-janz@uiowa.edu; 5 Institute for Cancer Genetics, and Department of Pathology and Cell Biology, College of Physicians & Surgeons, Columbia University, New York, NY 10032, USA; E-Mails: dc723@columbia.edu (D.C.); wg8@columbia.edu (W.G.); 6 Department of Clinical Oncology, Queen Elizabeth Hospital, Hong Kong, China; E-Mail: chocs@ha.org.hk

**Keywords:** ARF-BP1, B-cell lymphoma, p53, MYC, CTCF, ARF

## Abstract

Transcriptional activation of MYC is a hallmark of many B cell lineage neoplasms. MYC provides a constitutive proliferative signal but can also initiate ARF-dependent activation of p53 and apoptosis. The E3 ubiquitin ligase, ARF-BP1, encoded by *HUWE1*, modulates the activity of both the MYC and the ARF-p53 signaling pathways, prompting us to determine if it is involved in the pathogenesis of MYC-driven B cell lymphomas. ARF-BP1 was expressed at high levels in cell lines from lymphomas with either wild type or mutated p53 but not in ARF-deficient cells. Downregulation of ARF-BP1 resulted in elevated steady state levels of p53, growth arrest and apoptosis. Co-immunoprecipitation studies identified a multiprotein complex comprised of ARF-BP1, ARF, p53, MYC and the multifunctional DNA-binding factor, CTCF, which is involved in the transcriptional regulation of MYC, p53 and ARF. ARF-BP1 bound and ubiquitylated CTCF leading to its proteasomal degradation. ARF-BP1 and CTCF thus appear to be key cofactors linking the MYC proliferative and p53-ARF apoptotic pathways. In addition, ARF-BP1 could be a therapeutic target for MYC-driven B lineage neoplasms, even if p53 is inactive, with inhibition reducing the transcriptional activity of MYC for its target genes and stabilizing the apoptosis-promoting activities of p53.

## 1. Introduction

Human Burkitt lymphoma (BL) is characterized in most cases by reciprocal chromosomal translocations that juxtapose the *MYC* proto-oncogene with immunoglobulin (Ig) heavy chain or light chain regulatory sequences resulting in deregulation of *MYC* transcription [1]. Since MYC is critically involved in both G0→G1 cell cycle entry and cell cycle progression, both mRNA and protein levels must be tightly regulated to prevent abnormal cell growth. Normally, both MYC mRNA and protein exhibit rapid turnover with protein instability manifested by a half-life of less than 30 min [2]. Increased levels of MYC protein are reported in many cancers and have, in some instances, been associated with extended half-lives for BL and pediatric lymphoblastic lymphoma (LL) [2,3]. High levels of MYC protein in cancers may thus reflect impairment of degradation pathways as well as increased transcription.

MYC stability and transcriptional activity are both affected by multiple posttranslational modifications including phosphorylation, acetylation and ubiquitylation that serve to integrate the input from multiple signaling cascades. At least four different E3 ligase complexes contribute to MYC ubiquitylation and proteasome-mediated degradation: SKP2, FBW7 [4–7], ARF-BP1/HUWE1/HECT9 [8], and the recently described TRUSS-DDB1-CULA complex [9]. In each instance, overexpression of a dominant negative form, knockdown or gene deletion led to decreased MYC turnover. A comprehensive model for how the activity of these complexes is assimilated to direct MYC transcriptional activity and protein stability in different types of normal cells or in cancers, including MYC-associated human BL and mouse MYC-driven lymphomas, has not been developed. Information on the features of each of these complexes has nonetheless been accumulating at an accelerated rate.

Recent studies showed that SKP2 is expressed at high levels in most BL as well as lymphomas of Eμ-MYC transgenic (TG) mice [10,11]. The mouse lymphomas are phenotypically similar to normal immature or transitional B cells and together with tumors of λ-MYC TG mice [12] have been classified as diffuse high-grade blastic B cell lymphoma/leukemia (DBLL) [13]. Increased expression of SKP2 in DBLL was shown to be MYC-dependent but indirect, involving transcriptional as well as posttranslational mechanisms [10]. SKP2 interacts with MYC at promoters, acting as a co-factor for transcriptional activation, but subsequently mediates polyubiquitylation and proteasomal degradation.

The action of FBW7 on MYC requires prior phosphorylation at Ser-62 as a prerequisite for GSK3-dependent phosphorylation at Thr-58. FBW7, recruited to MYC phosphorylated at Thr-58, polyubiquitylates MYC, branching through Lys-48, and leading to its proteasomal degradation. Although FBW7 has been considered as the primary determinant of MYC degradation, the finding that MYC protein levels are not enhanced by expression of stable Thr-58 mutants is inconsistent with this conclusion [14]. The potential contributions of FBW7 downregulation to the development of BL have not been explored.

The TRUSS-DDB1-CUL4 E3 ligase complex targets both MYC and MYCN for ubiquitylation and proteasomal degradation independent of MYC phosphorylation on Thr-58 [9]. TRUSS expression is reduced in tumor cells, suggesting that downregulation may promote tumor formation by enhancing MYC protein stability. However, the previous tumor survey did not include hematopoietic neoplasms and, more specifically, BL.

The transcriptional activity of MYC is enhanced by recruitment of the histone acetyl transferases (HATs) CBP/p300 to gene promoters. Subsequent binding of ARF-BP1 results in polyubiquitylation with Lys-63 branching which does not lead to degradation but lead to enhanced interaction with CBP/p300 and stimulation of MYC acetylation. ARF-BP1 has also been shown to ubiquitylate p53, thereby promoting its degradation [14–16]. These activities of ARF-BP1 are inhibited by binding to ARF [17]. Again, the potential role of ARF-BP1 in modulating MYC-activated pathways in B cell lymphomagenesis has not been investigated.

The current studies were undertaken to better understand the complex dynamics of ARF-BP1 and its partner proteins and targets in the transformation of B lineage cells by MYC, utilizing BL cell lines and cell lines derived from DBLL of MYC TG mice. Our study aims to support the hypothesis that by regulating MYC and p53 transcriptional activity, ARF-BP1 is a critical determinant of the proliferation of B cell lymphomas and suggest that interference with ARF-BP1 provides a potential strategy to inhibit MYC activity in these tumors.

## 2. Results

### 2.1. ARF-BP-1 Is Expressed at High Levels in MYC-Driven Human BL and Mouse DBLL

**Figure 1:**
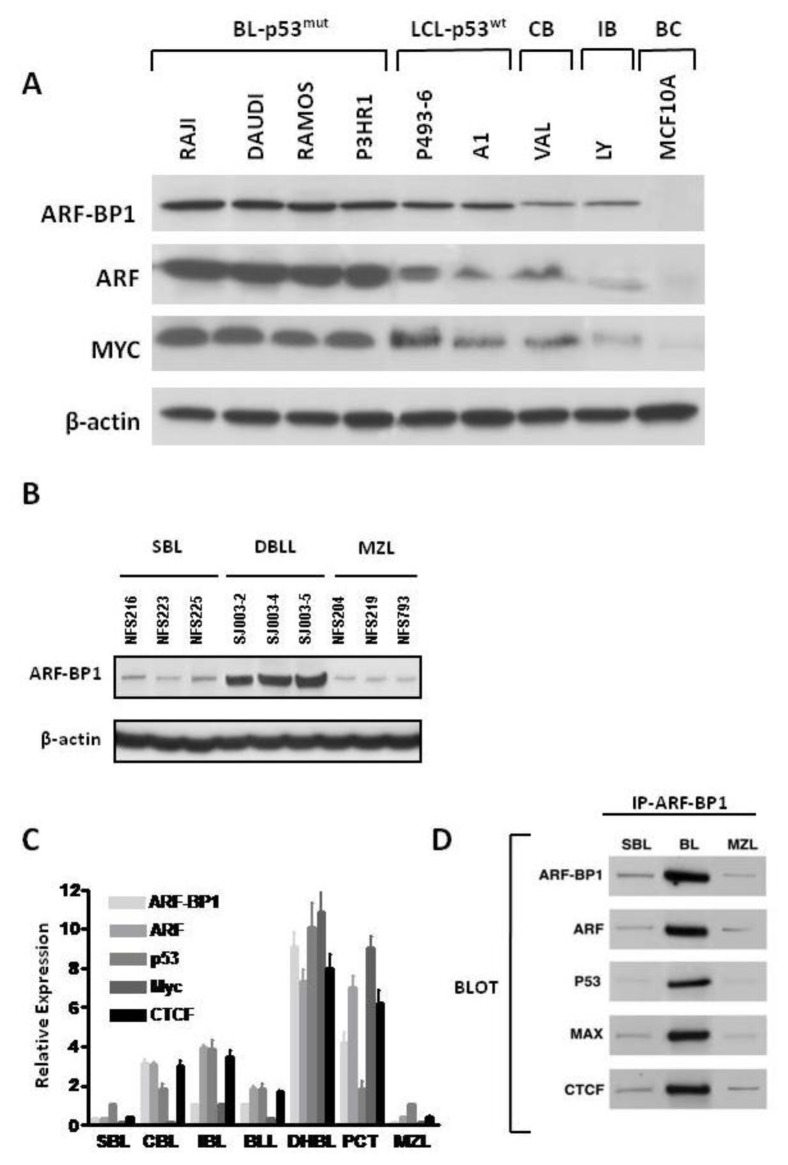
(**A**) Western blot analyses of ARF-BP1, ARF, and MYC in human Burkitt lymphoma (BL), EBV-transformed lymphoblastoid cells (LCL) and diffuse large B cell lymphomas (DLBCL). Mutational status of p53 in BL and LCL is indicated. MCF10A is a non-tumorigenic epithelial cell line as a negative control of ARF-BP1; (**B**) Western blot analyses of ARF-BP1 expressions in small B cell lymphomas (SBL-NFS216, NFS223 and NFS225), splenic marginal zone lymphomas (MZL-NFS204, NFS219 and NFS793) and λ-MYC diffuse high grade B cell lymphoblastic lymphomas (DBLL-SJ003-2, SJ003-4, and SJ003-5) from NFS.V^+^ mice; (**C**) qRT-PCR analyses of transcript levels for ARF-BP1, ARF, p53, MYC, and CTCF in cell lines derived from different classes of B cell lineage neoplasms; (**D**) Western blot analyses of cell lysates from SBL, λ-MYC TG DBLL and MZL-derived cell lines immunoprecipitated with ARF-BP1 antibody and blotted with antibodies to ARF-BP1, ARF, p53, MYC and CTCF.

Constitutive MYC-dependent activation of a large number of genes involved in a broad range of metabolic processes is responsible for the development of a variety of cancers [18,19]. Dosage-dependent effects of MYC on transformation are well established [20–22], and studies of primary human solid tumors have shown that levels of ARF-BP1 expression parallel the requirements for MYC in proliferation [23]. To examine the potential contributions of ARF-BP1 to MYC-driven B cell neoplasms, we elected to study cell lines derived from human BL and mouse DBLL from λ-MYC TG mice. We first examined the levels of ARF-BP1 protein expressed by BL cell lines mutant for p53, EBV-transformed lymphoblastoid cells (LCL) lines with wild type (wt) p53, centroblastic (CB) and immunoblastic (IB) diffuse large B cell lymphomas (DLBCL), and the epithelial cell line, MCF 10A (Figure 1A). These studies showed that ARF-BP1 was expressed at higher levels by the BL cell lines than by the DLBCL lines and that MCF 10A cells were completely negative. In addition, it appeared that the levels of ARF-BP1 expression were higher in the BL lines bearing *p53* mutations than in the LCL with a wt *p53* gene. The relative protein levels of ARF and MYC to ARF-BP1 were nearly similar for each of the cell lines (Figure 1A). Parallel studies of mouse cell lines established from primary small B cell lymphomas (SBL), DBLL and splenic marginal zone lymphomas (MZL) showed that ARF-BP1 was expressed at uniquely high levels in MYC-driven DBLL (Figure 1B). These findings indicate that ARF-BP1 is expressed at high levels in human and mouse B lineage lymphomas that express MYC at high levels.

We next proceeded to determine the patterns of expression for ARF-BP1 and its established partner and target proteins, ARF, p53 and Myc, by qRT-PCR analyses of transcript levels in a large panel of primary B cell lineage neoplasms from NFS.V^+^ mice diagnosed as SBL, DLBCL of centroblastic (CBL) and immunoblastic (IBL) types, DBLL, and MZL (Figure 1C). This analysis includes plasmacytoma (PCT) cell lines from BALB/c mice as NFS.V^+^ PCT are infrequent. We also studied the same tumors for expression of CTCF, a versatile transcription factor previously shown to be involved in regulating the expression of ARF [24], p53 [25] and MYC [26]. The results showed that ARF-BP1 and ARF were expressed at the highest levels in cells over-expressing MYC from the λ-MYC TG or due to activating chromosomal translocations in PCT. These results are consistent with earlier studies indicating that MYC contributes to the transcriptional activation of ARF. There is currently no basis for understanding why transcript levels of ARF-BP1 levels are elevated in cells with deregulated MYC, although earlier studies indicated that ARF-BP1 is not a direct transcriptional target of MYC [17].

Previous studies demonstrated protein-protein interactions between ARF-BP1 and ARF, MYC, p53 and MCL1 with some of these interactions being context-dependent. For example, access of ARF-BP1 to MCL1 is reported to occur only in cells exposed to DNA damaging agents [27]. Interestingly, CTCF has been shown in unpublished studies to interact physically with MYC [28]. These findings prompted us to ask if ARF-BP1 might interact with CTCF. We immunoprecipitated ARF-BP1 from SBL, DBLL and MZL-derived cell lines, separated the proteins by SDS-PAGE, and blotted with antibodies to ARF-BP1, ARF, p53, MYC and CTCF (Figure 1D). Immunoprecipitation with normal IgG served as a negative control. The results showed that, as expected, ARF, p53 and MYC co-immunoprecipitated with ARF-BP1 from lysates of the DBLL cell line that expresses ARF-BP1 at high levels. Strikingly, a strong signal was also obtained for CTCF, identifying it as another component of this macromolecular complex. All four partner proteins were also brought down, but at lower levels, in precipitates from the SBL and MZL cell lines (Figure 1D). The IP controls including protein-A-agarose beda, IgG and antibody against beta-actin that does not bind with ARF-BP1, did not produce bands when blotted with antibodies against ARF, ARF-BP1, p53, MYC, and CTCF (data not shown). Further studies would be required to determine which proteins in this complex interact directly with the others and which are pulled down through indirect interactions.

### 2.2. ARF-BP1 Binds to and Ubiquitylates CTCF

Previous studies demonstrated that the functional activity of CTCF is modulated by phosphorylation [29]. In addition, SUMOylation contributes to the repressive function of CTCF on the *MYC* P2 promoter [30] and poly-ADP ribosylation is reported to be important for CTCF-dependent chromatin insulation [31]. The finding that CTCF and ARF-BP1 associate *in vivo* under pathologic conditions suggested that CTCF might also be modified by ARF-BP1-dependent ubiquitylation. To investigate this possibility, we performed studies using full length CTCF or the central CTCF zinc finger (ZF) region and showed that both constructs pulled down ARF-BP1 (data not shown).

**Figure 2:**
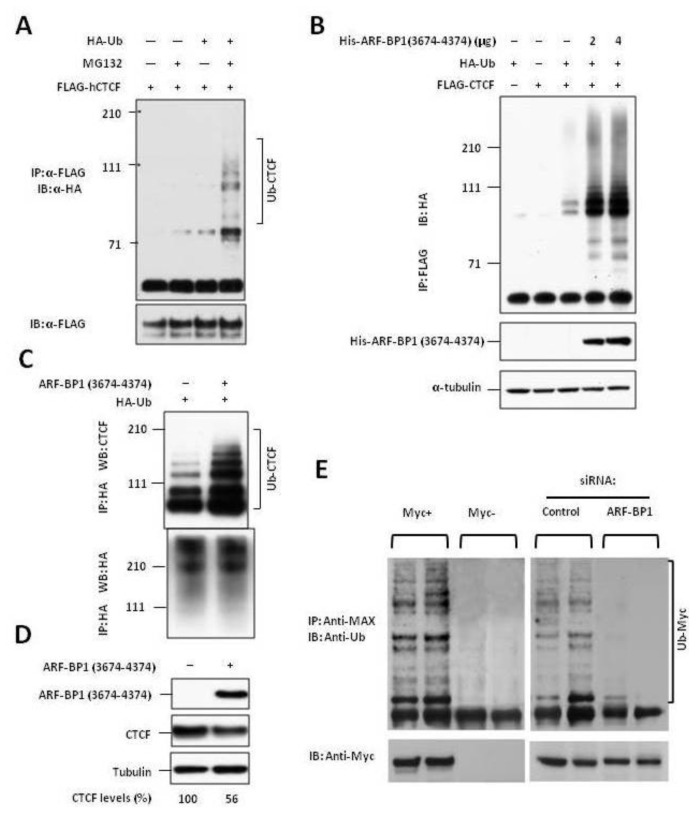
(**A**) 293 cells were co-transfected with FLAG-CTCF and with HA-tagged ubiquitin (HA-Ub) in the presence or absence of MG132. Proteins were precipitated with anti-FLAG and blotted with anti-HA; (**B**) Overexpression of ARF-BP1 (3674-4374) enhances polyubiquitylation of CTCF. FLAG-tagged CTCF, HA-Ub, and an increasing amount of His-ARF-BP1 (3674-4374) were cotransfected in 293T cells as indicated. Proteins were precipitated with anti-FLAG and blotted with anti-HA; (**C**) ARF-BP-1 ubiquitylates endogenous CTCF. HA-Ub and His-ARF-BP1 (3674–4374) were co-transfected into 293T cells in the presence of MG132 for 6 h and then harvested and lysed. Proteins were precipitated with anti-HA and blotted with anti-CTCF monoclonal antibody (D31H2); (**D**) CTCF levels are reduced in cells overexpressing ARF-BP1. HA-Ub and FLAG-ARF-BP1 (3674-4374) or vector alone was transfected in 293T cells. After 24 h, the cells were harvested and lysed. Proteins were precipitated with anti-FLAG and blotted with anti-HA. CTCF levels were quantitated by densitometry; (**E**) Using IP and co-IP methods, MYC ubiquitylation was detected in BL cells (left), and ARF-BP1 binds MYC and induces its ubiquitylation (right). ARF-BP1-mediated ubiquitylation of MYC in BL is inhibited by ARF-BP1 siRNA (right).

Although previous studies had shown that CTCF was post-translationally modified by the small ubiquitin-like protein SUMO with the repressive polycomb protein, Pc2, acting as a SUMO E3 ligase, there are no reports of CTCF modification by ubiquitylation [30]. To determine if CTCF can be ubiquitylated *in vivo*, 293 cells were co-transfected with FLAG-CTCF and HA-tagged ubiquitin (HA-Ub) in the presence or absence of the proteasome inhibitor, MG132. The 293 cells were used rather than B cell lines because of technical limitations posed by efficient transfection of non-adherent cells. Cell lysates were precipitated with anti-FLAG. The precipitates were separated electrophoretically and immunoblotted with anti-HA-tag to detect ubiquitylated CTCF. The results of these studies (Figure 2A) demonstrated that CTCF when overexpressed could be modified by ubiquitin and that polyubiquitylated forms were readily detected in cells blocked for proteasomal degradation.

To determine if ARF-BP1 was capable of acting as an E3 ligase for CTCF, we co-transfected 293 cells with FLAG-CTCF, HA-Ub and increasing amounts of His-tagged C-terminal region of ARF-BP1 that contains the HECT domain, aa 3674–4374. Protein lysates were precipitated with anti-FLAG and the precipitates separated electrophoretically and immunoblotted with anti-HA to detect ubiquitylated CTCF. The results (Figure 2B) showed that overexpression of ARF-BP1 resulted in enhanced polyubiquitylation of CTCF in a dose-dependent manner, identifying ARF-BP1 as an E3 ligase for CTCF.

We then asked if ARF-BP1 might be active in ubiquitylating endogenous CTCF. 293 cells treated with MG132 were transfected with Ha-Ub and His-ARF-BP1 (3674–4374) and lysed 6 h later. HA-Ub-modified proteins were immunoprecipitated with anti-HA mAb, and then analyzed for the extent of CTCF ubiquitylation by immunoblotting with a mAb to CTCF. These studies (Figure 2C) showed that endogenous CTCF was constitutively ubiquitylated and that the levels of modification were greatly increased in cells overexpressing ARF-BP1.

Our earlier studies suggested that when CTCF was overexpressed, ubiquitylation directed the protein to proteasomal degradation. To determine if ubiquitylation by ARF-BP1 would affect the stability of endogenous CTCF, 293 cells were transfected with empty vector or vector expressing His-tagged ARF-BP1 (3674–4374). The cells were harvested 24 h later and endogenous CTCF levels were determined by immunoblotting and quantified by densitometry using tubulin levels as a control (Figure 2D). The results showed that the levels of endogenous CTCF were reduced by nearly 50% in cells overexpressing ARF-BP1. We conclude that ARF-BP1 is an E3 ligase for endogenous CTCF and that polyubiquitylation of CTCF leads to its proteasomal degradation.

### 2.3. ARF-BP1 Ubiquitylates MYC in B Lineage Cells

Previous studies using U2OS, HeLa and 293 cells demonstrated that ARF-BP1 catalyzes K-63-linked ubiquitylation of MYC thereby switching MYC from a repressive to an activating state [23]. To determine if MYC is ubiquitylated by ARF-BP1 in B cell neoplasms, we first took advantage of the P493-6 human B cell line that is derived from an EBV-immortalized B cell line and carries a conditional tetracycline-regulated *MYC* [24]. Protein extracts from cells with MYC “on” (Myc+) and Myc “off” (Myc−) were precipitated with antibodies to its transcriptional partner protein, MAX, separated by SDS-PAGE and blotted with antibodies to ubiquitin or MYC (Figure 2E, left panel). The results revealed multiple bands reactive with the anti-Ub antibodies in the extracts from MYC “on” cells but not MYC “off” cells, demonstrating that MYC is highly ubiquitylated as in other cell types. To determine if ARF-BP1 was responsible for MYC ubiquitylation, we performed similar studies with MYC+ cells that had been treated with an ARF-BP1-specific or a control siRNA (Figure 2E, right panel). The results showed that, as previously established for other cell types [23], MYC ubiquitylation in B lineage cells was highly dependent on ARF-BP1.

### 2.4. Inactivation of ARF-BP1 Stabilizes p53, Induces p53-Dependent Apoptosis, and Reduces Cell Proliferation in λ-MYC TG Cells

**Figure 3:**
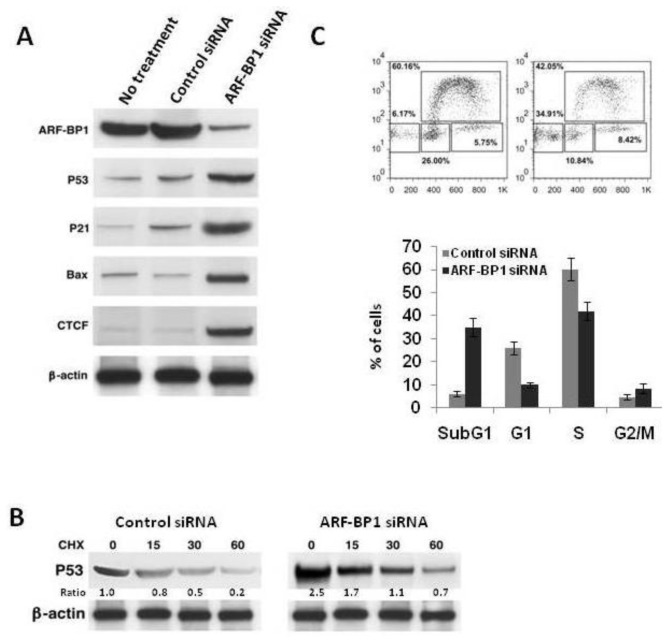
Effects of ARF-BP1 downregulation on expression of p53, p21, Bax and CTCF and cell cycle progression. (**A**) Protein extracts from DBLL cells with wt p53 transfected with an ARF-BP1-specific or a control siRNA were blotted with antibodies to the indicated proteins; (**B**) Quantitation of p53 protein levels in cells transfected with a control or an ARF-BP1-specific siRNA relative to levels of β-actin, indicated by ratios; (**C**) Cell cycle analysis of DBLL cells treated with a control siRNA or an ARF-BP1-specific siRNA by flow cytometry and quantitation of the frequencies of sub-G1 apoptotic cells and cells in G1, S or G2/M.

We next examined the effects of changes in ARF-BP1 levels induced by suppressive siRNA on expression of p53, the p53 transcriptional targets, p21 and BAX, and on CTCF (Figure 3A). A DBLL cell line derived from a λ-MYC lymphoma that was treated with an ARF-BP1-specific siRNA had greatly increased levels of each of these proteins, consistent with studies performed with cells from other lineages [15,17]. The effects of ARF-BP1 suppression on CTCF were in keeping with our earlier studies indicating that ubiquitylation enhanced proteasomal degradation of the protein. Interestingly, the enhanced levels of p53 protein associated with ARF-BP1 knockdown were not associated with increased protein stability, suggesting important contributions from other degradative and presumably ARF-BP1-dependent mechanisms (Figure 3B).

The changes in expression of p53, p21 BAX and CTCF induced by siRNA to ARF-BP1 were reflected by changes in cell cycle regulation and apoptosis. As shown in Figure 3C, cells treated with the ARF-BP1-specific siRNA had reduced frequencies of cells in cycle and had greatly increased numbers of sub-G1 cells, indicative of an apoptotic population.

## 3. Discussion

The functions of the p53 tumor suppressor protein and the *MYC* oncogene are finely tuned through a myriad of interactions with other proteins. These interactions can lead to posttranslational modifications that regulate protein stability, DNA binding or promoter-specific transcriptional activation or repression [14,23,32]. It is currently well accepted that ubiquitylation plays a major part in regulating the activities of both p53 and MYC and that this reflects the activities of a seemingly ever increasing number of different E3 ligases [25,32]. ARF-BP1 is unique among these E3 ligases in that it targets both p53 and MYC with profound effects on the development and growth of a variety of MYC-driven solid tumors [15].

Firstly, the results presented here demonstrated that ARF-BP1 was also highly expressed in MYC-dependent B cell lineage neoplasms of both humans and mice. Interestingly, the mouse neoplasms that expressed ARF-BP1 at high levels derived from near opposite ends of the mature B cell developmental spectrum in mice including DBLL that derive from pre-germinal center (pre-GC) B cells to PCT, which originate from mature plasma cells. In humans, they also included BL and DLBCL of GC origin.

Secondly, depletion of ARF-BP1 in a DBLL cell line resulted in inhibition of cell cycle progression and increased apoptosis. These results paralleled to previous studies of ARF-BP1 knockdowns in *MYC*-dependent human tumor cell lines raising the possibility that ubiquitylation of MYC may be the defining function of ARF-BP1 in tumor survival and proliferation [15]. Importantly, ARF-BP1 was expressed in human tumors and LCL expressing either wt or mutant p53 supporting the suggestion that it could be a therapeutic target in a variety of MYC-dependent lymphomas or EBV-dependent lymphoproliferative disorders regardless of p53 status [17].

Thirdly, while previous studies had shown that ARF, ARF-BP1 and MYC could form a ternary complex [26], our analyses of DBLL cells demonstrated that ARF-BP1 could be part of an even larger multiprotein complex that also includes p53, MAX and CTCF. The presence of p53 and MAX in this complex can probably be understood as a consequence of p53 being a known target of ARF-BP1 and the partnership of MYC and MAX in MYC transcriptional activation promoted by ubiquitylation of MYC by ARF-BP1.

**Figure 4:**
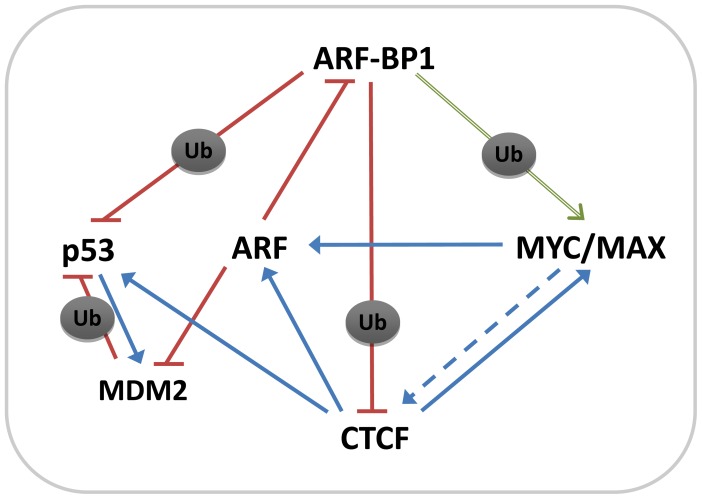
Features of protein-protein interactions and transcriptional regulation of genes involved governing cell proliferation and survival of MYC-driven B cell lineage neoplasms. Post-translational ubiquitylation (Ub) of p53 and CTCF by ARF-BP1 and p53 by MDM2 is inhibitory (red lines and bars), directing proteasomal degradation. Ubiquitylation of MYC by ARF-BP1 promotes transcriptional activity of MYC/MAX heterodimers (green arrow). Binding of CTCF to the promoters of p53, ARF and MYC is permissive for transcription while MYC effects transcriptional activation of ARF and p53 activates transcription of MDM2 (blue arrows). ARF inhibits the activities of both ARF-BP1 and MDM2 (red bars). Previous studies also indicate that MYC can indirectly promote transcriptional activation of CTCF.

The identification of CTCF as part of this complex is of particular interest for several reasons; the first is its contributions to regulated expression of other protein components of the complex (Figure 4). Binding of CTCF to the *p53* promoter is reported to protect the gene from epigenetic silencing [33], while methylation-sensitive binding to sequences in the regulatory region is required for activation of the *INK4A/ARF* locus [34]. Secondly, binding of CTCF at the human *MYC* locus is required for gene expression and protection from methylation [35]. Finally, ectopic expression of CTCF results in profound inhibition of cell cycle progression [36,37], phenotypes we found to be associated with depletion of ARF-BP1.

The interactions of ARF-BP1and CTCF were shown to require the central 11 ZF DBD of CTCF and the C-terminal region of ARF-BP1 that contains the HECT E3 ligase domain. Studies using transfected CTCF showed that it was polyubiquitylated, the levels of ubiquitylation were increased following co-transfection with an ARF-BP1 expression vector and the effect of ubiquitylation was to direct modified CTCF to proteasomal degradation. Finally, we showed that polyubiquitylation was not dependent on overexpression of CTCF and the levels of endogenous CTCF ubiquitylation were increased in cells co-transfected with ARF-BP1.

Previous studies showed that CTCF interacts with a wide range of proteins that may approach 60 in number and include YY1, PARP1, NPM, UBF, cohesins, and RNAPII among others [32,38]. Although CTCF was previously shown to be modified post-translationally by SUMOylation [30], phosphorylation [39] and poly(ADP) ribosylation [31], to our knowledge this is the first report that CTCF is modified by ubiquitylation. While the other modifications affect the functional activity of CTCF, they have not been reported to alter its stability. Currently, we do not know if ARF-BP1 is the only E3 ligase capable of modifying CTCF, the nature of ubiquitylation, or if ubiquitylation is countered by the activity of deubiquitylating enzymes such as DUB1 [32]. It will be very important to determine the sub-cellular localization of the ARF-BP1-CTCF interaction. If it occurs in the nucleus, it may counteract SUMOylation. Since SUMOylated CTCF inhibits MYC promoters, ubiquitylation may result in transcriptional activation at these target sites.

CTCF is a highly versatile protein that serves a wide variety of functions [40]. At present, it is unsure whether these functions might be affected by changes in CTCF levels along with cell cycle progression, stress or differentiation or by differences in subcellular localization of CTCF.

Ubiquitylation plays a key role in regulating the activity of multiple oncoproteins, including those of the MYC family. MYC is a short-lived protein that is degraded through the ubiquitin-proteasome pathway [41]. The F Box E3 ligase, FBW7, recognizes MYC phosphorylated at threonine 58 by GSK3, polyubiquitylates MYC, and then leads its degradation by the 26S proteasome [42]. Inhibition of GSK3 stabilizes MYC in lymphoma patients [43]. However, ubiquitylation of transcription factors does not necessarily result in inhibition, although it can control their activity independent of proteasomal degradation [44]. It has been reported that the switch between transcriptional activation and repression by MYC is regulated by site-specific ubiquitylation [23]. SKP2 is another E3 ligase for MYC ubiquitylation, binding to a conserved sequence element in the amino-terminus [23]. This is thought to be essential for transformation and transcriptional regulation [4].

It has been shown that ARF-BP1 also functions as an E3 ubiquitin ligase for MYC and it regulates the switch between the activated and repressed state of the MYC protein. Inactivation of ARF-BP1 repressed MYC ubiquitylation in λ-MYC cells that express a high level of activated MYC. Previous studies demonstrated that ARF-BP1 assembles Lys63-linked polyubiquitin chains on MYC and this modification is required for gene activation by MYC, allowing the interaction of MYC with the p300 coactivator [23]. Such K63-linked polyubiquitin chains do not target proteins for proteasomal degradation; instead they regulate the function of the modified protein. However, a recent report has shown that ARF-BP1 ubiquitylates MYCN through Lys48-mediated linkages and thereby targets it for destruction by the proteasome [8]. The induction of ARF by MYC resulting in inhibition of ARF-BP1 may serve as a negative feedback loop to limit excessive MYC function.

Recent support for the concept that ARF-BP1 is critically important in regulating the balance between pathways governing survival and death of B cell lineage lymphomas, in part through effects on p53, comes from studies of mice with a B cell-specific deficiency in ARF-BP1 [16]. Mutant B cells exhibited elevated expression of p53 and of p53 target genes in association with impaired development and homeostasis. Similar conclusions regarding ARF-BP1 control of p53 activity were drawn from studies of pancreatic B cells conditionally deficient in expression of ARF-BP1 [45].

As summarized in Figure 4, the studies presented here identify a previously unrecognized component of an interacting network of proteins that control proliferation and apoptosis in MYC-driven B cell lineage lymphomas of mice and humans. To the well-documented interactions between ARF-BP1, MYC, p53 and MDM2, established from studies of a variety of solid tumors, we have now added the multifunction nuclear factor, CTCF, and showed that it is part of the scaffolding of a multimolecular complex. The effects of CTCF on the transcription of MYC, ARF and p53 are certainly nucleoplasmic and the ARF-BP1-CTCF interactions are equally certain to be nuclear and directly chromatin-associated as there is little or no cytoplasmic CTCF and almost all CTCF appears to be tightly chromatin bound. It will be of interest to determine if all CTCF is equally accessible to ARF-BP1 as it is in nucleus during interphase, associates with the centrosome during mitosis and localizes to the midbody and reformed nuclei during telophase [29,31,32,38,40]. How ubiquitylation relates to other post-translational modifications will also be of considerable interest. Finally, it remains to be determined if the sole effect of ARF-BP1-mediated ubiquitylation on CTCF is to direct proteasomal degradation.

## 4. Experimental Section

### 4.1. Mice and Cell Lines

NFS.V^+^ mice that develop a spectrum of B cell lineage neoplasms [16] and λ-MYC TG mice were described previously [12,46]. Tissues obtained at necropsy were used for tumor classification using established criteria [47], and for later preparation of RNA, DNA and protein extracts. Plasmacytoma (PCT) cell lines were a gift from Michael Potter (NCI, NIH, Bethesda, MD, USA). All animal studies were performed under NIAID IACUC approved protocol LIP6.

Single cell suspensions prepared from spleen or lymph nodes of tumor bearing λ-MYC TG mice were cultured to develop cell lines. The cell lines were shown to be clonal based on Southern blot analyses of immunoglobulin heavy chain organization and were found to be phenotypically similar to normal splenic transitional B cells [48].

Human BL and EBV-transformed LCL cell lines were generously provided by G.W. Bornkamm [49] and M.D. Scharff (BL2). Human diffuse large B cell lymphoma (DLBCL) cell lines VAL and LY were a gift from R. Dalla-Favera. MCF 10A, and HEK 293 cell lines are from our laboratories.

### 4.2. Protein extraction, Western Blotting, and Immunoprecipitation

Nuclear and cytoplasmic protein fractions were extracted and western blotting was performed as previously described [50]. For immunoprecipitation studies, 300 μg of protein in 500 μL of 2X immunoprecipitation buffer (2% Triton X-100, 300 mM NaCl, 20 mM Tris [pH 7.4], 2 mM EDTA, 2 mM EGTA, pH 8.0, 0.4 mM sodium orthovanadate, 0.4 mM PMSF and 1.0% NP-40) and 400 μL H_2_O were incubated with anti-ARF-BP1 or other antibodies on beads (NeoMarkers, Fremont, CA, USA), according to the manufacturer’s instructions. The immune complexes were collected and analyzed by SDS-PAGE.

### 4.3. RNA Isolation and Analysis by Quantitative RT-PCR (qRT-PCR)

Total RNAs were isolated from tumor cells and used for qRT-PCR as described previously [50]. Primers designed using the Primer Express software (Applied Biosystems, Foster City, CA, USA). All samples were tested in triplicate. The comparative C_T_ method was used for quantification of gene expression. Statistical analysis was performed using SDS v2.1 software (Applied Biosystems) according to the manufacturer’s instructions. *Gapdh* was used as an endogenous reference.

### 4.4. Ubiquitylation Assay

For detection of protein ubiquitylation, cells were washed with ice-cold PBS and lysed in a NP40-containing buffer (50 mM Tris-HCl [pH 7.4], 250 mM NaCl, 5 mM EDTA, 50 mM NaF, 1 mM Na_3_VO_4_, 1% NP40, 0.02% NaN_3_, 1 mM PMSF, and protease inhibitor cocktail [Roche]) for 30 min on ice. Extracted proteins were pre-cleared with protein G agarose beads (Invitrogen) followed by incubation with 5 μg Ab coupled to protein G agarose beads overnight at 4 °C. Beads were washed three times in 20 vol of ice-cold lysis buffer, resuspended in 1× NuPAGE LDS sample buffer (Invitrogen) containing 50 nM DTT, and boiled for 5 min. Equal amounts of protein for each sample were separated on 3–8% Tris-Acetate gels (Invitrogen) and subsequently transferred to PVDF membranes (Invitrogen) in 1X NuPAGE transfer buffer (Invitrogen). After blocking with 5% skim milk for 1 h in PBS-T (0.1% Tween 20 in PBS), membranes were subsequently incubated with primary Abs and then with respective HRP-conjugated secondary Abs (Santa Cruz). Blots were developed with either SuperSignal West Pico or Dura (Pierce) and exposed to Biomax MR film (Kodak).

The antibodies used in this experiment were mouse monoclonal antibodies specific for Flag (1:1000; M2; Sigma), His (1:500; Invitrogen) and rat monoclonal antibody specific for HA (1:1000; Roche).

### 4.5. Cell Cycle Analysis

The Click-iT EdU Flow Cytometry Assay Kit (Invitrogen) was used to analyze cell cycle, according to the manufacturer’s instructions.

## 5. Conclusions

Taken together, the present findings demonstrated that ARF-BP1 is an important determinant in MYC-driven lymphoid malignancies of mice and humans by virtue of its interactions with ARF, p53 and MYC, as well as CTCF (Figure 4). ARF-BP1 can act to promote BL tumor development by: (1) inducing ubiquitylation and degradation of p53; (2) enhancing the transcriptional activity of MYC; and (3) by direct ubiquitylation of CTCF. Down-regulation of ARF-BP1 could be an important therapeutic target for MYC-driven B cell lineage neoplasms, especially for cases with p53 aberrations, by reducing transcriptional activation of MYC target genes. ARF-BP1 may thus provide a potential target for developing improved treatments for human BL.
